# 环境样品中双酚类化合物的固相萃取研究进展

**DOI:** 10.3724/SP.J.1123.2021.02035

**Published:** 2021-08-08

**Authors:** Hongyuan LIU, Jing JIN, Cuicui GUO, Jiping CHEN, Chun HU

**Affiliations:** 1.中国科学院分离分析化学重点实验室, 中国科学院大连化学物理研究所, 辽宁 大连 116023; 1. CAS Key Laboratory of Separation Science for Analytical Chemistry, Dalian Institute of Chemical Physics, Chinese Academy of Sciences, Dalian 116023, China; 2.沈阳药科大学, 辽宁 沈阳 110000; 2. Shenyang Pharmaceutical University, Shenyang 110000, China; 3.中国科学院大学, 北京 100012; 3. University of Chinese Academy of Sciences, Beijing 100012, China

**Keywords:** 双酚类化合物, 固相萃取, 分子印迹, 固相微萃取, 磁固相萃取, bisphenol compounds, solid phase extraction (SPE), molecular imprinting, solid phase microextraction (SPME), magnetic solid phase extraction (MSPE)

## Abstract

双酚类化合物作为一类内分泌干扰物广泛存在于环境介质中,经过多种途径迁移至人体后,可对人体产生内分泌毒性、细胞毒性、基因毒性、生殖毒性、二噁英毒性和神经毒性,已被加拿大政府风险评估识别为进一步优先控制名录。随着环境领域对双酚类化合物的广泛关注,相关研究工作逐渐向水、沉积物、灰尘和生物样品等多介质开拓。但是,由于不同环境样品在基质复杂性和污染物浓度水平等方面存在显著差异,开发提取效率高、净化选择性好、普适性强、操作简单、高通量的提取和净化方法,有助于实现环境介质中双酚类化合物的高灵敏、批量检测。近年来,新型前处理技术发展迅速,尤其是固相萃取技术,在双酚类化合物提取与净化方面取得了长足的发展,不仅在一定程度上克服了传统提取净化方法存在的耗时、耗力和耗溶剂等不足,而且为新型污染物分析提供了更多的技术支持。该文简述了典型双酚类化合物的理化性质、用途用量和环境危害,重点围绕新型固相萃取吸附剂开发和固相萃取模式转变两个方面,总结了固相萃取在双酚类化合物提取净化方法方面取得的进展。商品化固相萃取产品普适性强,在环境监测领域应用范围较广,适用于双酚类化合物的产品种类有限;新型吸附剂研发聚焦吸附容量(如介孔硅材料、碳纳米材料、金属-有机框架材料、环糊精)和选择性(如分子印迹聚合物和混合模式离子交换聚合物)两个方面,种类多样化可满足不同检测需求;越来越多的高灵敏分析仪器不断推向市场,为适应新的发展形势,固相萃取模式正逐渐向微型化、自动化、简易化等方向发展,如QuEChERS、固相微萃取、磁固相萃取等。

## Abstract (en)

Owing to the strict restrictions on the production and use of bisphenol A (BPA), bisphenol analogs (e. g., bisphenol S and bisphenol F) are gradually coming to use in many fields. BPA and these bisphenol analogs are so-called bisphenols (BPs). BPs as a class of endocrine disrupters are widely distributed in the environment (water, sediments, sludge, and aquatic products). BPs enter the human body through various routes, leading to endocrine disruption, cytotoxicity, genotoxicity, reproductive toxicity, dioxin-like effects, and neurotoxicity. The Canadian government has identified BPs as substances for further scoping/problem formulation. Because of the widespread attention paid to BPs in the environmental field, research is being expanded to cover water, sediment, dust, and biological samples, and other media. Given the significant differences in the complexity and pollution concentration of environmental samples, the development of pretreatment methods that afford high extraction efficiency, good purification selectivity, strong universality, operational simplicity, and high-throughput extraction and purification, are necessary to realize the highly sensitive detection of BPs in environmental media. In recent years, solid-phase extraction (SPE), accelerated solvent extraction (ASE), microwave-assisted extraction (MAE), and dispersion liquid-liquid-microextraction (DLLME) as new pretreatment technologies have gradually replaced the traditional liquid-liquid extraction and Soxhlet extraction. SPE has seen rapid development for the extraction and purification of BPs in various environmental samples, overcoming the bottlenecks related to time, energy, and solvent consumption in traditional methods while extending technical support for the analysis of emerging pollutants. The physicochemical properties, usage, and environmental hazards of typical BPs were briefly reviewed, with emphasis on the application of SPE products, development of new adsorbents, and transformation of the SPE mode. Commercialized SPE products are universally applicable in the field of environmental monitoring, while products suitable for the pretreatment of BPs are limited. The development of new adsorbents mainly focused on their adsorption capacity and selectivity. For example, ordered mesoporous silicon, carbon nanomaterials, metal-organic frameworks, and cyclodextrins have large surface areas, good adsorption performance, and regular pore structures, which improve the adsorption capacity of BPs. Molecularly imprinted polymers (MIPs) and mixed-mode ion-exchange polymers are mainly used to improve the selectivity of BPs in the purification process. In addition, MIPs have high chemical, mechanical, and thermal stabilities, which ensures their widespread application in the extraction, preconcentration, and separation of BPs. A variety of new SPE adsorbents can partially meet the diverse needs for detection. There is a consensus that the current challenges in analytical chemistry include the determination of contaminants at low concentration levels, but at the same time, more efficient and environment-friendly methodologies are required. With the introduction of high-sensitivity instruments in the market, the SPE model is seeing gradual development in terms of miniaturization, automation, and simplification. This in turn has minimized solvent consumption, analysis time, and labor cost, resulting in more efficient and affordable analytical methods such as QuEChERS, solid-phase microextraction (SPME), and magnetic solid-phase extraction (MSPE) to adapt to the new development scenario.

双酚类化合物是一类含有两个酚羟基且结构相似的化学物质,是合成高分子材料的重要化工原料之一。其中,以双酚A(bisphenol A, BPA)在国内外的使用最为广泛,它主要用于聚碳酸酯(占全球BPA总产量的近70%)和环氧树脂(占全球BPA总产量的近30%)等高分子聚合物的生产。由于聚碳酸酯和环氧树脂具有高性能、可持续和生态高效等特点,通常用于生产可重复使用的水瓶和食物储存容器、光盘、眼镜、医疗设备、建筑材料、容器涂层以及高性能油漆和涂料等^[1]^。据全球市场调研,2015年BPA的全球消费量约为770万吨,预期到2022年将达到1060万吨^[2]^。人体生物监测研究表明,BPA广泛存在于人类日常生活环境中,大约近九成美国人尿液中可检测出BPA^[3]^。BPA低水平长期暴露会对人类健康产生影响,与肥胖^[4]^、癌症^[5]^、糖尿病^[6,7]^、男性生殖功能障碍^[8]^以及心血管^[9]^等疾病的产生具有相关性。2010年起加拿大政府和欧盟委员会已经发布相关法规禁止制造、进口和销售含BPA的聚碳酸酯婴儿奶瓶^[10,11]^。

随着法规对BPA的严格限制,用作BPA替代品的双酚类似物相继出现。加拿大政府已经识别并将包含双酚F(bisphenol F, BPF)、双酚S(bisphenol S, BPS)、双酚B(bisphenol B, BPB)、双酚E(bisphenol E, BPE)、双酚AF(bisphenol AF, BPAF)、双酚Z(bisphenol Z, BPZ)等在内的34种双酚类化合物进行进一步风险管控^[12]^。目前,双酚类化合物的生产和应用在全球范围内都呈现增长趋势,许多双酚类似物表现出内分泌干扰作用、细胞毒性、基因毒性、生殖毒性和神经毒性,其中BPAF、BPB、BPF和BPS表现出与BPA相似甚至更高的雌激素作用^[13]^,可能会对人体的内分泌、生殖和神经等系统产生影响。典型双酚类化合物的理化性质见表1,结构见图1。

**表1:** 典型双酚类化合物的理化性质^[14]^

Chemical	CAS No.	IUPAC name	Formula	*M*_r_	log *K*_ow_	p*K*_a_
BPA	80-05-7	4-[2-(4-hydroxyphenyl)propan-2-yl]phenol	C_15_H_16_O_2_	228.29	3.32	9.60
BPB	77-40-7	4-[2-(4-hydroxyphenyl)butan-2-yl]phenol	C_16_H_18_O_2_	242.31	4.13	10.10
BPC	79-97-0	4-[2-(4-hydroxy-3-methylphenyl)propan-2-yl]-2-methylphenol	C_17_H_20_O_2_	256.35	4.74	9.86
BPS	80-09-1	4-(4-hydroxyphenyl)sulfonylphenol	C_12_H_10_O_4_S	250.27	1.65	8.20
BPAF	1478-61-1	4-[1,1,1,3,3,3-hexafluoro-2-(4-hydroxyphenyl)propan-2-yl]phenol	C_15_H_10_F_6_O_2_	336.23	4.47	9.20
BPF	620-92-8	4-[(4-hydroxyphenyl)methyl]phenol	C_13_H_12_O_2_	200.23	2.91	7.55-10.80
BPE	2081-08-5	4-[1-(4-hydroxyphenyl)ethyl]phenol	C_14_H_14_O_2_	214.25	3.19	10.10
TBBPA	79-94-7	2,6-dibromo-4-[2-(3,5-dibromo-4-hydroxyphenyl)propan-2-yl]phenol	C_15_H_12_Br_4_O_2_	543.87	7.20	8.50
BPAP	1571-75-1	4-[1-(4-hydroxyphenyl)-1-phenylethyl]phenol	C_20_H_18_O_2_	290.35	4.86	10.22
BPZ	843-55-0	4-[1-(4-hydroxyphenyl)cyclohexyl]phenol	C_18_H_20_O_2_	268.35	5.00	9.76-10.37

log *K*_ow_: octanol-water partition coefficient; p*K*_a_: acidity coefficient.

**图1:**
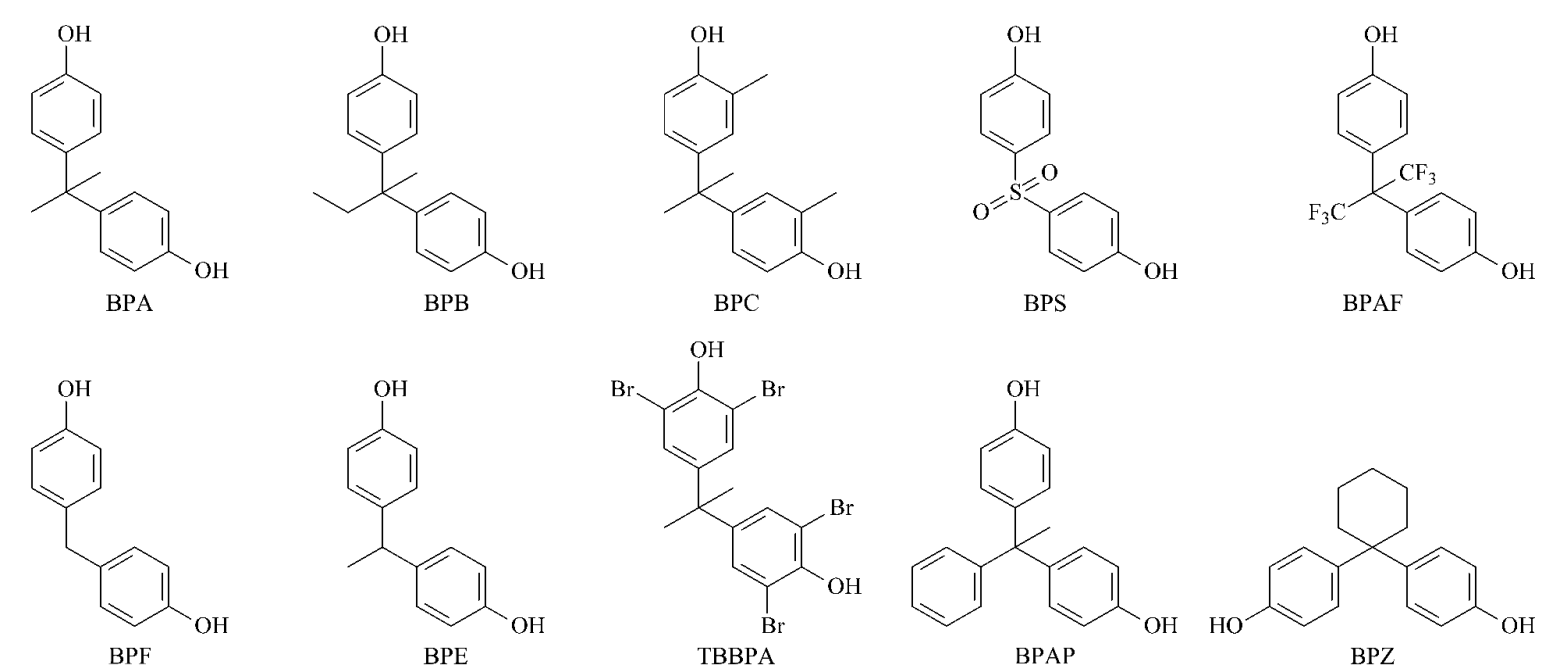
典型双酚类化合物的结构式

双酚类化合物主要存在于地表水、沉积物、室内灰尘和生物样品等复杂的环境介质中。到目前为止,环境样品中能够检测到的BPs主要有BPA、BPF、BPB、BPS、BPE和BPAF。研究发现:环境样品中BPs含量较低,且污染物种类间浓度差异较大,复杂基质的干扰对样品检测具有较大影响^[15,16,17]^。这一客观事实对环境样品中双酚类化合物的前处理方法开发提出了较高的要求。传统的液液萃取和索氏提取效率较高,但是耗时、耗力、耗溶剂,逐渐被固相萃取、加速溶剂萃取、微波辅助萃取等技术所替代。由于固相萃取可集提取、分离、浓缩三位为一体,具有操作简单、萃取吸附剂种类多样、环境相对友好、易于实现自动化等特点,在环境多介质(水体、土壤、沉积物、灰尘等)检测领域得到广泛应用^[18]^。本文重点围绕新型固相萃取吸附剂和新型固相萃取模式,介绍固相萃取技术在环境样品中双酚类化合物提取与净化方面的研究进展。

## 1 常规固相萃取(SPE)

硅基吸附剂作为常用的固相萃取产品,由于残存活性羟基在极端条件下的不稳定性,通常对其表面进行改性,比如十八烷基硅烷(C18)等^[19,20]^。随后出现了石墨化炭黑(GCBs)和多孔石墨碳(PGCs)等碳基吸附剂,这些吸附剂对某些化合物的保留作用很强,甚至是不可逆的。聚合物基吸附剂的出现克服了这些缺点,实现了形态和化学性能的有机结合,可与多种类型的化合物产生适当的相互作用,并保持较强的稳定性。环境介质中常用于双酚类化合物检测的聚合物吸附剂是二乙烯基苯/*N*-乙烯基吡咯烷酮共聚物(HLB)^[21,22,23,24,25]^。Yang等^[21]^对比了HLB、C18和ENVI-Carb石墨化炭黑对水体中双酚类化合物的提取效果,结果表明:C18小柱对BPS和BPF的萃取效率最低(回收率<20.00%),这是由于C18功能团具有疏水性,对极性化合物的保留作用较弱;GCB小柱对BPS、TCBPA和TBBPA的萃取效率相对较低(回收率<60.00%),这是由于GCB被视为阴离子交换剂,酸性较强的化合物会与阴离子交换剂结合,难以洗脱下来;HLB对于7种双酚类化合物表现出最佳萃取效果(回收率85.40%~105.8%),且应用最为广泛。

固相萃取产品的推广和使用提高了环境样品的前处理效率,在一定程度上促进了我国环境污染物监测工作的顺利进展。同时,商品化固相萃取产品由于受吸附剂种类的限制,尚无法完全满足环境科学研究对新型有机污染物前处理的特殊要求。

## 2 新型固相萃取

鉴于环境介质中双酚类化合物存在浓度低、化学性质差异大、基质复杂的特点,研究工作对目标物的吸附容量或选择性提出更高要求,进而推动了新型吸附材料的研发进程。诸如介孔硅材料^[26,27,28]^、碳纳米材料^[29,30,31]^、金属有机骨架(MOFs)^[32,33,34]^和环糊精材料^[35,36]^等主要用于提高对目标物的吸附容量(见表2);而分子印迹聚合物(molecularly imprinted polymers, MIPs)和混合模式离子交换聚合物(mixed-mode ion-exchange polymers)的研发主要用于提高对目标物的选择性富集或净化。

**表2:** 新型固相萃取吸附剂在环境样品中双酚类化合物高效富集中的应用

Sorbent	Analytes	Samples	Measurement	Recoveries/%	Ref.
MI-SBA-15	BPA	tap/well/waste water	HPLC	87.00-110.2	[27]
MI-SMS	BPA, BPF, BPB, BPE, BPAF	sediment	HPLC-DAD	75.50-105.5	[28]
COF-GO	BPA	river/sea water	CFDI-MS	95.30-106.6	[29]
COOH-MWCNT	TBBPA, BPA	lake/sea water	LC-MS	82.00-99.00	[30]
HPCSs	BPS, BPF, BPA, BPC, TBBPA, BPAP, BPAF, TCBPA	river water	HPLC-DAD	89.60-111.5	[31]
MOF/CS/PEO foam	BPA, BPB, BPC, BPAF, BPF	tap water	HPLC	72.68-104.6	[32]
Zr(Ⅳ)-MOF	BPA, BPB, BPAF, BPFL, BPS, BPF	tap water	LC-MS/MS	-	[33]
MP-CDP	BPA, BPF, BPAF	drinking water	HPLC	92.90-107.0	[35]

MI-SBA-15: molecularly imprinted ordered mesoporous silica SBA-15; MI-SMS: molecularly imprinted sponge mesoporous silica; COOH-MWCNT: carboxyl-functionalized multiwalled carbon nanotubes; COF-GO: covalent organic framework-graphene oxide; HPCSs: hollow porous carbon spheres; Zr(Ⅳ)-MOF: Zr(Ⅳ)-metal-organic framework; MOF/CS/PEO foam: metal organic framework/chitosan/polyethylene oxide foam; MP-CDP: microporous beta-cyclodextrin polymer; CFDI-MS: constant flow desorption ionization mass spectrometry.

### 2.1 提高吸附容量

2.1.1 有序介孔硅材料

有序介孔二氧化硅具有易功能化、吸附性能好、吸附动力学快等特点,常用吸附剂包括MCM41(Mobil Composition of Matter No. 41)、SBA(Santa Barbara amorphous)和六角介孔硅(HMS)。介孔二氧化硅的表面改性可进一步拓宽这些材料的物理和化学性质,提高其在分析化学领域的应用范围。比如,采用一锅法制备的十八烷基功能化有序介孔二氧化硅,比表面积高达796 m^2^/g,孔体积为0.88 cm^3^/g,可以作为反相固相萃取吸附剂应用于内分泌干扰物的净化^[26]^。同时,介孔硅材料表面具有典型的自组装介孔结构,被认为是替代有机聚合物制备分子印迹材料的理想选择。Zhang等^[27]^采用半共价法制备了分子印记有序介孔二氧化硅SBA-15(MI-SBA-15)材料,高度有序的介孔结构和较大的比表面积(643 m^2^/g)使其对BPA的吸附容量可达27.9 mg/g,对水样中BPA的回收率可达87.0%~110.2%。杨甲甲等^[28]^以卵磷脂/十二烷胺混合胶束为孔道模板,硅酸四乙酯为交联剂,通过异氰酸基团和BPA的酚羟基之间形成可逆的氨基甲酸酯键制备了海绵状的BPA印记介孔硅材料(SMS)。该材料不仅具有相互贯通的3D-孔道结构,比表面积(850.55 m^2^/g)和孔体积(1.20 cm^3^/g)较大,而且可以在3 min内选择性吸附BPA(38.6 mg/g),可用于底泥样品中5种BPs(BPA、BPF、BPB、BPE、BPAF)的净化,回收率在75.50%~105.5%之间。

2.1.2 碳纳米材料

碳纳米材料指的是分散相中至少有一维在纳米尺度的碳材料,包括富勒烯(C60)、碳纳米管、石墨烯、介孔碳等。C60具有球形结构,表面积大,但其在水和有机溶剂中的溶解度低,在分析化学中的应用较少。碳纳米管具有较高的机械稳定性和热稳定性,对其表面进行有机基团修饰可提高其吸附/解吸能力。Kou等^[30]^比较了C60、多壁碳纳米管(MWCTN)及其羧基功能化纳米管(COOH-MWCNT)3种不同碳材料作为固相萃取吸附剂对水体中TBBPA和BPA的吸附效率,COOH-MWCNT吸附率可达到80.00%以上。三维多孔石墨烯材料是以石墨烯片层为组成单元的三维多孔组装体,具有大比表面积、高机械强度和高效的传质效率等优良特性。与其他碳质材料相比,石墨烯在表面化学修饰方面具有明显优势。石墨烯通过剥离后可获得氧化石墨烯,其表面存在羟基、羧基等含氧基团,易于在其表面插入其他反应性化学基团,从而获得其他选择性石墨烯基吸附剂。Gao等^[29]^制备了一系列共价有机骨架-氧化石墨烯复合材料,其中三甲酰基间苯三酚/联苯胺/氧化石墨烯复合物(TpBD-GO-2)对BPA表现出的吸附能力分别比单一材料高2.2倍和4.7倍。该材料可应用于河水和海水中BPA的富集,回收率达95.30%~106.6%。Zhang等^[31]^通过扩展的一步Stöber方法制备了一种新型球体,经过碳化和模板去除工艺,合成了相对均匀的单分散空心多孔碳球(HPCSs)。与MWCNT和3D-石墨烯相比,HPCSs具有三维纳米结构,比表面积更高(571.278 m^2^/g)、分散性更好,对于环境水样中BPs(BPS、BPF、BPA、BPC、TBBPA、BPAP、BPAF、TCBPA)的富集效率可达89.60%~111.5%。

2.1.3 金属有机骨架材料

金属有机骨架是由过渡金属团簇和有机配体组成的多孔配位聚合物,能形成三维有机-无机杂化网络,主要类型包括沸石咪唑骨架(ZIFs)、莱瓦希尔骨架(MILs)等。尽管它们有不同的性能,但总的来说,均具有比表面积大、孔道结构规则、孔隙率高等性能^[37]^。但是由于MOFs粒径较小且通常表现为非球形,因此,它们一般需要与不同的载体相结合,以便作为SPE材料应用于双酚类化合物的富集。Yu等^[33]^使用Zr(Ⅳ)基金属-有机骨架(BUT-17)富集水体中的双酚类化合物,对BPA的吸附容量为111 mg/g,吸附速率较快(1.76 g/(mg·min))。Li等^[32]^将不同MOF材料分散在含冰醋酸的超纯水中,加入壳聚糖、聚环氧乙烷和明胶,连续搅拌下加入5%戊二醛,将此混合物注入立方硅橡胶模具中,开发了金属有机骨架/壳聚糖/聚环氧乙烷泡沫(MOF/CS/PEO)新型材料。其中,MIL-53(Al)制备的泡沫材料MIL-53(Al)/CS/PEO具有许多隧道结构,比表面积(383.05 m^2^/g)更大、吸附效率更高,可富集环境水样中72.68%~104.6%的BPs(BPA、BPB、BPC、BPAF、BPF)。彭俊钰等^[34]^采用逐步络合生长法制备了可控纳米级ZIF-8@SBA-15有序介孔-微孔复合材料,该材料具有高比表面积(722 m^2^/g)和高吸附容量(135.1 mg/g)。该复合材料的协同作用使其在水中对BPA的吸附容量和吸附速率分别是单一ZIF-8的2倍和20倍,吸附平衡时间较短(2 min左右)。

2.1.4 环糊精材料

*β*-环糊精(*β*-CD)是一种由7个葡萄糖单元组成的环状低聚糖,具有疏水性内腔和亲水性外表面,其对疏水性有机分子具有分子识别能力^[38]^。Li等^[35]^采用十氟联苯作为交联剂制备了一种新型微孔*β*-CD聚合物(MP-CDP),表面积为261.1 m^2^/g,孔径约为1.1~1.7 nm,对3种双酚(BPA、BPF、BPAF)均表现出较高的吸附能力(最大吸附量为78.93 mg/g)。MP-CDP对环境水体中3种双酚的富集效率高达92.90%~107.0%,且重复使用性好,萃取效率和富集系数高。Cai等^[36]^以四氟对苯二甲腈为交联剂制备了羟丙基-*β*-环糊精(HP-*β*-CD)的多孔聚合物,该材料对BPA、BPS和BPF具有优异吸附性能,不仅吸附容量可分别达到99.01、63.29和68.03 mg/g,而且吸附速率很快,1 min内BPA就可达到吸附平衡,10 min内BPS和BPF可达吸附平衡。另外,在5次吸附/解吸循环后,HP-*β*-CD聚合物的吸附性能几乎没有下降,具有可重复使用性。

### 2.2 提高选择性

2.2.1 分子印迹聚合物

MIPs是通过模拟酶与底物或抗原抗体特异性结合原理,以某一特定的目标分子为模板,在功能单体和交联剂存在下制备的对目标分子具有特定选择性的聚合物。分子印迹技术具有预定性、识别性和实用性等特点,已被广泛应用于样品前处理方面^[39]^。模板分子和功能单体之间的相互作用力和结合位点的数量是决定MIP高选择性的因素之一,为获取更多结合位点,双重位点印迹策略被提出,可实现两个模板分子之间高亲和力结合。因此,越来越多的双模板或多模板MIP被开发出来。然而,由于使用目标化合物作为模板分子存在模板分子渗漏问题,严重影响目标化合物的准确定量,所以替代模板分子印迹技术(相关片段、同位素标记的化合物或目标分子的其他结构类似物)逐渐发展为环境分析领域解决模板泄露最安全通用的方法^[40]^。下文将逐一介绍已报道的适用于双酚类化合物前处理的分子印迹聚合物。

2.2.1.1 单模板分子印迹聚合物

Dong等^[41]^通过4-乙烯基吡啶(VP)和*N*-异丙基丙烯酰胺(NIPAM)双官能单体的协同作用,制备了多孔载体上的热敏分子印迹聚合物(T-MIPs),通过温度调节来选择性识别和控制BPA的吸附和释放,并将其作为新吸附剂用于海水中BPA的提取,提取效率可达94.83%~98.47%。即使经过6次吸附-解吸循环后,回收率仍可达到90.00%,表明该材料具有较高的吸附性能和良好的稳定性。Kalogiouri等^[42]^通过溶胶-凝胶基质印迹技术合成具有高效、高选择性的新型BPA溶胶-凝胶MIP,对河水中BPA的萃取效率可达93.40%±0.90%。Lyu等^[43]^利用BPA作为模板,4-乙烯丙烯酸(4-VP)作为功能单体,乙烯乙二甲基丙烯酸酯(EDMA)作为交叉连接剂制备了离子液体介质分子印记聚合物(IL-MIP),该聚合物具有高吸附能力(116.16 mg/g)和高选择性,并将其用于湖水中BPA的测定,回收率为93.67%~102.1%。

2.2.1.2 双/多模板分子印迹聚合物

Xie等^[44]^采用表面压印技术即在介孔二氧化硅包覆的磁性氧化石墨烯(MGO@mSiO2)的表面上制备了一种新型的多模板分子印迹聚合物。该材料对BPA在内的烷基酚具有良好的吸附选择性,不仅可重复使用,而且对环境水样中BPA的富集回收率达到81.54%~106.7%。Wang等^[45]^以氢苯甲酸乙酯(EP)、BPA和邻苯二甲酸二丁酯(DBP)作为模板分子,EDMA为交联剂,2,2-偶氮二异丁腈(AIBN)为引发剂合成了三模板(TMIP)、双模板(DMIP)和单模板(SMIP)印迹聚合物,比表面积分别为228.29、233.57和206.86 m^2^/g。其中,以EP和DBP作为双模板的印迹聚合物表现出最佳的提取性能,对河水样品中BPA的回收率达到87.00%~120.0%。

2.2.1.3 替代模板分子印迹聚合物

Sun等^[46]^以BPAP为替代模板,4-VP为功能单体,乙二醇二甲基丙烯酸酯(EGDMA)和AIBN为交联剂和引发剂,通过皮克林乳液聚合的方法合成了分子印迹聚合物,该聚合物形状规则,粒径大小适合,具有高比表面积(355.759 m^2^/g)和高吸附能力(3.327 μmol/g),并将它应用于环境沉积物中7种双酚(双酚A、B、F、E、S、Z、AF)的选择性萃取,回收率为75.50%~105.2%。在此基础之上,还提出了一种基于非印迹色谱柱的替代模板和聚合物组成筛选方法,即采用非印迹材料作为液相色谱柱固定相,采用致孔剂作为流动相,模拟重现预聚合溶液中模板-单体相互作用情况,快速筛选了一系列用于双酚印迹的替代模板,如BPS、1,1,1-三(4-羟基苯基)乙烷(THPE)和酚酞(PP)^[47,48]^。以BPS、THPE和PP为替代模板分子,4-VP为功能单体,EGDMA为交联剂,乙腈为致孔剂制备了3种分子印迹聚合物(BPS-DMIP、THPE-DMIP和PP-DMIP),可用于环境样品中BPF、BPE、BPA、BPB和BPAF等双酚类化合物的选择性萃取,萃取效率大于89.00%^[49]^。其中,THPE-DMIP印迹材料对双酚类结构类似物(己烯雌酚、双烯雌酚等干扰物质)选择性净化效果明显。

综上,分子印迹固相萃取技术在双酚类化合物选择性富集/净化中的应用见表3。

**表3:** 分子印迹固相萃取技术在双酚类化合物选择性富集/净化中的应用

Sorbent	Analytes	Samples	Measurement	Recoveries/%	Ref.
Alternative template MIP	BPA, BPB, BPAF, BPAP, BPS,	river/tap water	HPLC	89.40-102.0	[49]
	BPF, BPE, BPZ				
Thermosensitive MIP	BPA	seawater	HPLC	94.83-98.47	[41]
Alternative template MIP	BPA, BPB, BPAF, BPAP, BPS,	sewage, sludge	HPLC	82.20-101.0 (sewage),	[48]
	BPF, BPE, BPZ, TBBPA			43.60-96.70 (sludge)	
MGO@mSiO_2_@MIP	BPA	water	HPLC	81.54-106.7	[44]
Alternative template MIP	BPA	water	HPLC	65.56-88.84	[40]
IL-MIP	BPA	lake water	UV-vis	93.67-102.1	[43]
sol-gel MIP	BPA	river water	HPLC	93.40±0.90	[42]
Dual template MIP	BPA	river water	HPLC	87.00-120.0	[45]

MIP: molecularly imprinted polymers; MGO@mSiO_2_@MIP: mesoporous silica coated magnetic graphene oxide MIP; IL: ionic liquid.

2.2.2 混合模式离子交换聚合物

混合模式离子交换高分子材料将具有非特异性相互作用的聚合物骨架与具有特异性相互作用的离子交换基团进行有机结合,可实现离子型或可离子型化合物的选择性富集或净化。主要包括强阳离子交换剂(SCX)、强阴离子交换剂(SAX)、弱阳离子交换剂(WCX)和弱阴离子交换剂(WAX)。SCX和WCX吸附剂通常分别由磺酸基和羧酸功能化;SAX吸附剂通常含有季铵基,而WAX通常含有叔胺、仲胺或伯胺。Lee等^[50]^将冻干的污泥样品用5 mL甲醇和水混合溶剂萃取60 min,用MCX柱对淤泥提取液进行净化,淤泥样品中BPs(BPA、BPAF、BPAP、BPB、BPF、BPP、BPS、BPZ)的回收率为(55.9±12.7)%~(157.0±9.6)%。Zhao等^[51]^采用MCX柱对海水和沉积物样品中的BPs(BPA、BPS、BPF、BPAF、BPB、BPP、BPFL)进行富集或净化,回收率分别为103.0%~178.0%和55.7%~116%。

## 3 QuEChERS

QuEChERS (quick, easy, cheap, effective, rugged, safe)作为一种快速、简单、价廉、高效、耐用及安全的前处理技术,是传统液-液萃取和固相萃取的替代方式。该技术早期只适用于水果蔬菜中农药的检测,现如今可以适用于农产品、环境和生物样品等基质中有机化合物的前处理^[52]^。操作流程如下:1)使用乙腈或酸化乙腈对均质后的样品进行提取;2)添加盐诱导相分离进行液-液分配;3)利用特定吸附剂进行分散固相萃取除去干扰物质,并用无水硫酸镁(MgSO_4_)脱水,促进目标化合物分配到有机层,同时加入氯化钠(NaCl)减少共萃取物,促进相分离^[53,54,55,56]^。最常使用的吸附剂主要是伯仲胺(PSA)和C18^[57]^(见表4)。Cerqueira等^[55]^在此基础上开发了新的QuEChERS方法,即使用从虾壳废料中获得的几丁质作为一种新型吸附剂,BPA回收率在87.00%~101.0%之间。与PSA和C18相比较,该吸附剂不仅可获得满意的净化效果,还节省了大量成本。

**表4:** QuEChERS在双酚类化合物前处理中的应用

Procedure	Analytes	Samples	Measurement	Recoveries/%	Ref.
Extraction: 10 mL ACN, 4 g MgSO_4_+1 g NaCl	BPA	fish	HPLC-MS/MS	80.80-118.6	[53]
Clean-up: 25 mg PSA+25 mg C18+2% NaCl					
Extraction: 10 mL ACN (1% HCl), 4 g MgSO_4_+1 g NaCl	BPA,	fish	LC-MS/MS	67.00-107.0	[54]
Clean-up: 500 mg C18+500 mg PSA+1500 mg MgSO_4_	TBBPA				
Extraction: 10 mL ACN(1% acetic acid), 4 g MgSO_4_+1 g NaCl	BPA	sludge	LC-MS/MS	87.00-101.0	[55]
Clean-up: 150 mg MgSO_4_+50 mg Chitin					
Extraction: 10 mL ACN (0.1% acetic acid)+5 mL water, 1 g MgSO_4_+1 g NaCl	EDCs	sediment	GC-MS/MS	60.00-130.0	[56]
Clean-up: C18+PSA	(BPA)				
Extraction: 4 mL ACN+2 mL saturated NaCl solution	TBBPA	aquatic	UPLC-MS/MS	74.00-121.0	[57]
Clean-up: 50 mg MgSO_4_+50 mg C18		products			

EDCs: endocrine disrupting chemicals.

## 4 固相微萃取(SPME)

固相微萃取通常是以高聚合物材料涂覆的石英玻璃纤维为吸附介质,可集萃取、浓缩、解吸和进样于一体^[58]^。作为一种环境友好的前处理技术,SPME已经成功应用于环境介质中酚类化合物的提取。以碳基骨架为基础改性的SPME纤维可用于提高目标物的吸附能力和选择性。新涂层材料包括离子液体、聚合物、氮和酸化合物的碳纳米管和石墨烯等。Braun等^[59]^研究了聚丙烯酸酯(PA)、聚二甲基硅氧烷(PDMS)和聚二甲基硅氧烷/二乙烯基苯(PDMS/DVB) 3种不同的SPME纤维涂层对双酚A等物质的萃取效率。PA和PDMS/DVB主要用于萃取酚类等极性化合物,PDMS纤维可用于富集更多非极性化合物,在最佳的提取条件下,PA是最有效的涂料。Wang等^[60]^采用基于多壁碳纳米管(MWCNTs)的SPME, 10 min内实现了水体中BPs的高倍浓缩和富集,提取效率在85.30%~102.5%之间。Frankowski等^[61]^将木质素磺酸盐掺入到聚3,4-乙撑二氧噻吩(PEDTO)结构中,制备了聚(3,4-乙撑二氧噻吩)/木质素磺酸盐电聚合吸附剂(PEDOT/LS),增强了材料对双酚的吸附能力,回收率在73.80%~102.8%之间(BPS除外)。该研究采用了弹簧形的支撑件代替了传统的直丝,提高了吸附剂的固载量,增大了对BPs的吸附容量。SPME技术克服了SPE存在的主要缺点,如萃取步骤多、溶剂量较多、不可重复使用等。除SPME外,管内固相微萃取、搅拌棒吸附萃取、注射器微萃取和分散固相萃取等小型萃取技术逐渐发展起来,在萃取和预浓缩步骤中大大减少了有机溶剂的消耗。

## 5 磁性固相萃取(MSPE)

磁性固相萃取是基于磁性或可磁化吸附剂的一种新型样品制备技术。与常规SPE相比,MSPE不需要使用固相萃取柱,具有萃取效率高、易于分离回收和可循环再利用等优点,在样品前处理技术中发挥着重要作用^[62]^。其中,铁氧化物在提取效率、富集因子、选择性和抗干扰能力方面表现突出,已经受到高度关注并被广泛应用^[63]^。通过对磁性四氧化三铁进行表面包覆或官能团修饰等方法,可以制备满足不同目的的MSPE吸附剂。目前,已报道的用于双酚类化合物富集净化的磁性材料包括沸石/氧化铁复合材料^[64]^、核壳碳修饰的磁性微球^[65]^、碳量子点/油酸包覆的Fe_3_O_4_复合材料^[66]^、氨基硅烷化磁性碳微球^[67]^、聚多巴胺涂层的磁性Fe_3_O_4_复合物^[68]^等。与石墨烯相比,氧化石墨烯表面有许多羟基、羧基和环氧基等基团,其与Fe_3_O_4_组成的复合材料为磁性固相萃取材料的研制提供了广阔的空间^[69,70]^,比如Fe_3_O_4_@SiO_2_/壳聚糖/氧化石墨烯/环糊精(MCGC)磁性复合材料^[71]^。Guo等^[72]^利用修饰基团的疏水性,制备了十二胺修饰的磁性氧化石墨烯吸附剂,用于富集环境水样中的BPA,提高了磁性材料对BPA的吸附效率,回收率达到74.90%~93.10%。在满足方法检出限的前提下,磁性固相萃取无疑将为提高样品中目标物的富集效率,简化操作流程提供可能。综上,磁固相萃取技术在水体中双酚类化合物前处理中的应用见表5。

**表5:** 磁固相萃取技术在水体中双酚类化合物前处理中的应用

Sorbent	Analytes	Samples	Measurement	Recoveries/%	Ref.
Fe_3_O_4_@PDA	BPF, BPA, BPB, BPAP	tap/pond water	HPLC	92.00-105.0	[68]
C-NH_2_@Fe_3_O_4_	BPA, BPAF, TBBPA	tap/slush/waste water	HPLC-MS/MS	86.10-110.0	[67]
Fe_3_O_4_/GO	BPA	waste water	HPLC	77.60-89.60	[69]
Fe_3_O_4_-OA/CQDs	BPA, BPAF, TBBPA	lake/waste water	FTIR	94.50-101.3	[66]
CMMPs	BPA, BPB, BPAF, BPF	tap/river water	UHPLC	85.40-104.0	[65]
HDTMA-ZSM-5/Fe_2_O_3_	BPA, BPAP, BPAF, BPP	tap/river/waste water	HPLC	83.00-108.0	[64]
MCGC	BPA, BPF	waste water	HPLC-FLD	90.20-97.70	[71]
MGO-DDA	BPA	lake/waste water	HPLC	74.90-93.10	[72]

Fe_3_O_4_@PDA: polydopamine coated magnetic Fe_3_O_4_ composite material; C-NH_2_@Fe_3_O_4_: aminosilanized magnetic carbon; Fe_3_O_4_/GO: magnetic Fe_3_O_4_ graphene oxide; Fe_3_O_4_-OA/CQDs: carbon quantum dots/oleic acid-coated Fe_3_O_4_ composites; CMMPs: Core-shell carbon decorated magnetic microspheres; HDTMA-ZSM-5/Fe_2_O_3_: hexadecyltrimethylammonium-zeolite/iron oxide magnetic composite; MCGC: Fe_3_O_4_@SiO_2_/chitosan/graphene oxide/*β*-cyclodextrin; MGO-DDA: dodecylamine modified magnetic graphene oxide nanomaterials.

## 6 总结

如何在低浓度水平上测定特定化学性质或不同化学性质的污染物是分析化学面临的挑战之一。在此基础上开发更有效、更加环境友好的分析方法是对分析化学的时代要求。因此,新型萃取产品和微型化提取技术得以开发,从而减少溶剂/试剂消耗,缩短分析时间,简化操作流程,产生更高效、更经济的分析方法。在此背景下,本文围绕近年来固相萃取吸附剂开发和固相萃取模式的革新,总结了环境样品中双酚类化合物的固相萃取技术进展。针对双酚类化合物的非靶标筛查,由于化合物之间的性质差异,主要适用于选择高吸附容量的非选择性吸附剂;而对于双酚类化合物靶标筛查而言,具有较高选择性的分子印迹聚合物则受到青睐。受益于分析检测仪器的发展,现代化检测设备灵敏度不断提升,固相萃取模式正逐渐向微型化、自动化、简易化等方向发展。可调杂化材料与微型前处理方法的结合将为以后双酚类化合物的研究打开新的窗口。
